# The London Hospitals' Arrangements

**Published:** 1914-08-29

**Authors:** 


### HOSPITALS AND THE WAR.

### The London Hospitals' Arrangements.

### ST. BARTHOLOMEW'S HOSPITAL.

*(From our Special Correspondent.)*

The governors have offered to place a wing of the hospital (198 beds), which was closed on August 1 for the ordinary periodical repairs, etc., at the service of the War Office in the event of additional accommodation being required for the sick or wounded. The wing will be staffed and maintained by the hospital.

The whole of the visiting medical and surgical staff belong to the Territorials, and No. 1 General Hospital R.A.M.C. (T.F.) (520 beds) is staffed entirely by St. Bartholomew's men.

Fifteen members of the resident staff and thirty students (dressers, etc.) have gone on active service with either the Navy or the Army, and a certain number of students have gone to the No. 1 Base Hospital.

The hospital has undertaken to provide twenty-eight sisters or nurses for the Admiralty and thirty for the War Office, and up to the present time thirty-five have been drafted to various naval or military hospitals.

In addition, some thirty members of the nursing staff belong to the Territorials and have been mobilised.

Certain re-arrangements have been rendered necessary in the working of the out-patient special departments, and until further notice they will be open only on two days a week, otherwise the service of the hospital will continue as usual, as it is recognised that the more urgent needs of the civilian population are not likely to be diminished.

### WEST HAM AND EASTERN GENERAL HOSPITAL.

*(From our Special Correspondent.)*

All the resident staff have left, mostly to take up duties connected with the war. Their places have been filled by locums, and the changes have been extremely numerous, but the work of the hospital has been kept going by the devotion of the honorary staff, who have given extra time in spite of the fact that many of them have also been more than usually occupied through the absence of partners. We have also lost several nurses, including the home sister, who has gone to the front. Some of the Red Cross workers from Essex have been allowed to attend the hospital to receive lessons in dressings and other nursing duties. We have also offered fifty beds for the use of the wounded should they be needed, which offer has been accepted by the Surgeon-General.

### THE LONDON.

*(From our Special Correspondent.)*

In view of the fact that it is likely that the Government will avail themselves of the 500 beds offered by the London Hospital, it has been decided for the present to admit only urgent and short-time cases, to clear the beds for the reception of the wounded. It is anticipated that some notice will be given as to when beds will be required, since the first wounded will naturally go to fill the regular Government hospitals. The above does not mean that 500 out of the total of 922 will simply be so allocated. The committee fully realise that the civil population has every claim to attention. At pressure the total accommodation can be increased to 1,150 beds. With 500 of these assigned to the sick and wounded, 650 would be available for the general public. If the stress was very great many more patients could be accommodated by adopting the expedient of erecting tents in the garden.

The work of the hospital is now practically going on as usual. During the summer months under ordinary circumstances many of the senior members of the staff are away. Their places are taken by assistant physicians and surgeons, who in their turn are relieved of out-patient work. The out-patient work is performed by the medical and surgical registrars. At the outbreak of the war there was a very natural desire on the part of the resident staff to serve their country in a manner more direct and active than by staying and attending to the sick at home. It was pointed out, however, that the hospital's obligations to the War Office and the Admiralty made it most desirable to keep a fairly full complement of qualified men at the hospital.

It was agreed that a certain number of the resident doctors might apply for posts under the War Office and Admiralty. Five under these conditions applied and were accepted. The house doctors drew lots for the five places. Some of the research work of the hospital in these exceptional times has had to be discontinued, and vacancies have been filled by doctors hitherto engaged in laboratory work.

With regard to the food contracts, the difficulties which threatened at the outbreak of the war have not matured so seriously as was expected. There is no war clause in the form of contract, and unless there is some such clause it is believed contractors are legally bound to supply goods, unless they can show (as is possible in certain articles of purely German origin) that it is physically impossible, owing to the war, to continue the supply. Amicable terms have been come to with contractors, who have shown every wish to deal fairly by the hospital, and in all probability a compromise will be made when the contracts cease, whereby the whole of the loss, owing to the rise in prices consequent on the war, shall not fall upon the contractors.

